# Evaluation of serum neutrophil gelatinase‐associated lipocalin in older patients with chronic kidney disease

**DOI:** 10.1002/agm2.12098

**Published:** 2020-03-27

**Authors:** Lulu Guo, Bei Zhu, Haichuan Yuan, Weihong Zhao

**Affiliations:** ^1^ Division of Nephrology Department of Geriatrics The First Affiliated Hospital with Nanjing Medical University Nanjing China

**Keywords:** aging, chronic kidney disease, neutrophil gelatinase‐associated lipocalin

## Abstract

**Objective:**

Chronic kidney disease (CKD) is a major health‐care burden all over the world, and aging is an important risk factor for end‐stage renal disease (ESRD). Neutrophil gelatinase‐associated lipocalin (NGAL) has been confirmed as a novel marker for early diagnosis of acute kidney injury. Other studies have found that NGAL takes part in the mechanisms of CKD progression. The aim of this study was to evaluate the expression of serum NGAL in CKD, particularly in elderly patients who rapidly progressed to end‐stage renal failure.

**Methods:**

Serum NGAL, cystatin C, creatinine, urea, and other factors were evaluated in a cohort of 160 CKD patients (mean age 75.29 ± 12.08 years) with various etiologies.

**Results:**

Serum NGAL was closely related to cystatin C, creatinine, urea, and estimated glomerular filtration rate (eGFR). Special correlations between NGAL and, respectively, anemia and hypoalbuminemia were also found. The cutoff value of NGAL was calculated from Stage 2 to Stage 5. Receiver–operator curve analysis showed good area under the curve (>0.8) and high sensitivity (> 70%) on the cutoff value of NGAL. The NGAL levels increased progressively with the increasing of 2‐ and 5‐year risk of ESRD using the Kidney Failure Risk Equations (KFRE).

**Conclusion:**

In elderly patients with CKD, serum NGAL reflects renal impairment and presents a strong and independent risk marker for progression of ESRD.

## 1 INTRODUCTION

Chronic kidney disease (CKD) is becoming a major and serious public health problem, leading to not only kidney damage but also multiple systemic disease. The prevalence of all CKD stages in adults varies worldwide from 7% to 12%.[1] The prevalence of CKD Stages G3‐G5 varies in different regions of the world, with values reported as 1.7% in China,[2] 3.1% in Canada,[3] 5.8% in Australia,[4] and 6.7% in the United States.[5] The mean age of the general population is increasing worldwide, resulting in the problem of more elderly patients.[6] In Europe, 28% of the population will be older than 65 years of age by the year 2060.[7] In the United States, by 2030, 20% of the population will be older than 65 years.[8] In China, the population of older than 60+ and 80+ years in 2050 will be 8 times than 1950.[9] Aging is strongly associated with an increased risk and incidence of acute kidney injury.[10] Statistics suggest that the prevalence of CKD in patients aged ≥ 70 years is twice the number of those aged < 60 years.[11]The growing number of older patients with an estimated glomerular filtration rate (eGFR) < 45 mL/min/1.73 m^2^ has resulted in an increased risk of cardiovascular disease (CVD) and all‐cause mortality; meanwhile, as kidney function further declines the progression to end‐stage renal disease (ESRD) requiring dialysis is incontrovertible.[12, 13, 14, 15, 16, 17] Early identification and intervention are two major measures to prevent further decline of renal function and delay adverse outcomes, such as non‐fatal CVD and mortality.[18] As tubular epithelial cells play a crucial role in the pathogenesis of CKD progression, a tubular injury marker—neutrophil gelatinase‐associated lipocalin (NGAL)—is expected to be useful in reflecting disease activity and kidney function. NGAL is a member of the lipocalin family; it has a small molecular weight stress protein and recent studies have focused on its special source releasing from injured renal tubular cells. Many studies show that NGAL could be a biological marker for kidney function.[19, 20, 21, 22, 23, 24]Because of their lower muscle mass, less physical activity, and reduced food intake, elderly people have lower creatinine (Cr) generation. Deviations in actual renal function are complicated not just by calendar age but also by physiological age, nutritional status, and frailty. As aging is associated with declining glomerular filtration rate (GFR), single factors, such as serum Cr or age alone, might be insufficient for estimation of GFR in older patients. So we aimed to evaluate whether NGAL could be a new biomarker in elderly patients with CKD.

## 2 MATERIALS AND METHODS

### 2.1 Study design and participants

We examined 160 Chinese patients with various degrees of renal impairment referred to the First Affiliated Hospital with Nanjing Medical University from January to June 2018. CKD was defined on the basis of repeated measures of eGFR determined using the Chronic Kidney Disease Epidemiology Collaboration (CKD‐EPI) equation.[13]

Inclusion criteria were patients older than 50 years and diagnosis of CKD at any stage. Exclusion criteria were patients with acute CVD (coronary artery disease, myocardial ischemia, cerebrovascular disease, or peripheral artery disease) in the past 3 months, uncontrolled hypertension and diabetes, infections requiring admission in the past 3 months, malignancy, liver or thyroid disease, organ transplantation, being on immunosuppressive treatment, and unwillingness to participate in the study. The data of 160 patients affected by different kidney diseases with various CKD categories were analyzed in the present study. To avoid interobserver differences, all patients were recruited by a single investigator.

The study was approved by the Institutional Ethics Committee of the First Affiliated Hospital with Nanjing Medical University and all participants provided consent before the testing. All procedures were in accordance with the ethical standards of the institutional and/or national research committee and with the 1964 Helsinki Declaration and its later amendments or comparable ethical standards.

### 2.2 Laboratory measurements

Blood and urine samples were taken in the morning before any food intake. In addition to NGAL, cystatin C (CysC) and other commonly tested biochemical parameters, such as urea, Cr, uric acid, serum electrolytes, albumin (ALB), hemoglobin, and proteinuria, were also tested in all patients according to standard methods in the clinical laboratory. The eGFR was calculated with the CKD‐EPI equation. The blood samples were centrifuged immediately at 1500 *g* and 4°C for 10 min, and the supernatants were stored at –80°C until further use. Ten milliliters of urine sample was mixed with 1 mL of 10 mM Tris buffer, pH8.6, with 0.05% Tween 20 and 0.01% NaN3 containing protease inhibitors (10 mM benzamidine, 10 mM aminocaproic acid, 20 mM ethylenediaminetetraacetate, and aprotinin). This mixture was centrifuged at 990 *g* for 5 min and stored at −80°C until assayed.

Serum NGAL was measured using quantitative fluorescence immunoassay (Getein Biotech, Inc. Nanjing, China). The intra‐assay coefficient of variation was 50 ng/L‐5000 ng/L and the linear correlation coefficient was *r* ≥ 0.990.

### 2.3 Statistical analysis

All statistical analyses were performed using SPSS 19.0. Non‐normally distributed variables were expressed as median with interquartile range, and normally distributed variables were as mean SD, as appropriate. Between groups, comparisons were assessed for nominal variables with the one‐way analysis of variance for the rest of the variables. Pearson’s correlation coefficient was used to determine correlations between variables. The diagnostic abilities of the tests were compared using the areas under the curves (AUCs). Receiver–operator curve (ROC) analysis was used to calculate the AUCs. The Youden index (sensitivity + specificity – 1)—an integrative indicator of sensitivity and specificity[25]—was used to determine the cutoff value for NGAL identifying the different CKD categories by the revised CKD classification. For all tests, *P* < 0.05 was used to assess the statistical results.

## 3 RESULTS

### 3.1 Clinical and laboratory data

One hundred and sixty patients (mean age 75.29 ± 12.08 years) with CKD Stages 1‐5 were included in this study. Demographic characteristics are shown in Table 1. The NGAL levels increased progressively with decreasing eGFR. There was also a significant trend and statistical difference between these groups in urea, Cr, CysC, calcium (Ca), phosphorus (P), ALB, high‐density lipoprotein cholesterol (HDL‐C), red blood count (RBC), hemoglobin (HGB), and hematocrit (HCT) (*P* < 0.05).

**Table 1:** Clinical and laboratory data of patients with CKD stratified by eGFR

Variable	All patients	eGFR					*P*
		>90	90‐60	60‐30	30‐15	<15	
n	160	10	51	38	32	26	
Age (years)	75.29 ± 12.08	55 ± 4.690*	74.13 ± 11.86	76.87 ± 11.08	80.52 ± 7.779	73.23 ± 13.00	<.0001
Sex, M/F	99/61	7/3	32/19	20/18	16/16	20/6	
BMI, kg/m2	21.8 ± 4.0	22.4 ± 5.3	21.7 ± 4.5	21.4 ± 3.7	20.8 ± 4.8	20.4 ± 3.2	.32
NGAL (ng/mL)	402.9 ± 285.2	157.6 ± 64.04	228.3 ± 127.3	346.9 ± 159.4	552.9 ± 323.5	729.5 ± 282.7	<.0001
CysC (mg/L)	3.038 ± 2.489	0.9230 ± 0.2486	1.367 ± 0.4260	2.776 ± 1.610	4.445 ± 1.971	5.771 ± 3.455	<.0001
eGFR (mL/mim/1.73 m^2^)	47.5 ± 28.06	94.54 ± 3.036	75.31 ± 8.281	44.06 ± 8.620	23.51 ± 3.905	10.60 ± 2.463	<.0001
Urea (mmol/L)	14.24 ± 9.377	5.724 ± 1.554	6.755 ± 2.557	12.67 ± 5.724	19.67 ± 6.197	26.82 ± 8.776	<.0001
Cr (μmol/L)	207 ± 195.7	65.00 ± 14.77	75.72 ± 19.62	132.8 ± 26.48	237.6 ± 51.46	585.7 ± 186.3	<.0001
UA (μmol/L)	350.6 ± 119.4	333.3 ± 53.62	318.8 ± 86.28	355.4 ± 147.7	345.4 ± 98.26	409.8 ± 150.7	.0906
Ca (mmol/L)	2.16 ± 0.1747	2.319 ± 0.1331	2.191 ± 0.1538	2.200 ± 0.1520	2.155 ± 0.1372	2.004 ± 0.2084	<.0001
Phos (mmol/L)	1.207 ± 0.3368	1.156 ± 0.1884	1.057 ± 0.1875	1.124 ± 0.2029	1.271 ± 0.3394	1.543 ± 0.4805	<.0001
ALT (U/L)	18.6 ± 14.94	24.29 ± 8.365	20.59 ± 15.57	15.92 ± 5.569	15.72 ± 12.72	17.70 ± 23.72	.3917
AST (U/L)	22.54 ± 17.70	22.57 ± 3.666	23.40 ± 14.45	21.49 ± 5.773	25.98 ± 34.03	18.27 ± 5.920	.6396
ALP (U/L)	98.22 ± 53.85	79.48 ± 23.98	88.53 ± 45.82	94.70 ± 26.31	103.5 ± 76.31	123.6 ± 66.66	.0886
GGT (U/L)	41.06 ± 65.09	24.43 ± 17.80	31.57 ± 27.24	37.01 ± 19.68	32.86 ± 26.20	53.38 ± 48.41	.4123
TC (mmol/L)	4.289 ± 1.153	4.809 ± 0.7166	4.322 ± 1.261	4.250 ± 1.248	4.410 ± 0.9596	4.085 ± 1.246	.6019
TG (mmol/L)	1.592 ± 1.567	1.387 ± 0.7574	1.833 ± 2.320	1.433 ± 0.7608	1.616 ± 1.249	1.480 ± 1.034	.8049
HDL‐C (mmol/L)	0.9985 ± 0.3106	1.263 ± 0.4109	1.043 ± 0.3243	1.030 ± 0.2978	0.9489 ± 0.2208	0.8235 ± 0.2602	.0025
LDL‐C (mmol/L)	2.797 ± 0.8249	3.178 ± 0.5335	2.716 ± 0.9717	2.775 ± 0.8628	2.969 ± 0.6507	2.750 ± 0.7946	.4895
LPa (mg/L)	376 ± 347.6	419.1 ± 392.8	321.0 ± 364.5	409.1 ± 391.6	427.4 ± 290.7	393.3 ± 328.6	.7153
TP (g/L)	62.19 ± 7.541	64.96 ± 5.790	61.20 ± 9.428	64.00 ± 8.401	62.24 ± 5.811	61.27 ± 4.090	.4167
ALB (g/L)	34.3 ± 5.119	39.72 ± 4.067	35.11 ± 6.225	34.36 ± 4.574	32.69 ± 3.164	32.47 ± 3.872	.0015
CRP (mg/L)	4.4 ± 1.5	3.4 ± 2.7	5.7 ± 1.9	4.0 ± 1.3	5.4 ± 1.7	4.6 ± 1.2	.56
RBC (10^12^/L)	3.567 ± 0.7640	4.470 ± 0.4642	3.955 ± 0.6736	3.774 ± 0.6556	3.048 ± 0.5585	3.071 ± 0.5866	<.0001
HGB (g/L)	108.1 ± 22.43	134.0 ± 14.05	119.6 ± 21.96	113.3 ± 17.00	92.59 ± 16.38	95.22 ± 15.97	<.0001
HCT %	32.65 ± 6.687	40.15 ± 4.082	36.18 ± 6.122	34.24 ± 4.889	27.74 ± 5.207	28.73 ± 5.061	<.0001
MCV (fL)	91.72 ± 5.992	90.77 ± 4.073	91.47 ± 6.468	91.12 ± 6.706	91.13 ± 4.601	94.01 ± 5.882	.3929
MCH (pg)	30.44 ± 2.189	30.45 ± 1.787	30.20 ± 2.546	30.12 ± 2.160	30.47 ± 1.469	31.21 ± 2.295	.419
MCHC (g/L)	331.9 ± 11.41	335.7 ± 7.146	330.0 ± 12.19	330.8 ± 10.77	334.6 ± 11.11	332.0 ± 12.40	.4829

Note

*P*‐values are for comparison across all six groups from one‐way analysis of variance.

Abbreviations: ALB, albumin; ALT, alanine aminotransferase; ALP, alkaline phosphatase; AST, aspertate transferase; BMI, body mass index; Ca, calcium; Cr, creatinine; CRP, C‐reactive protein; CysC, cystatin C; eGFR, evaluated glomerular filtration rate; GGT, gamma‐glutamyl transferase; HCT, hematocrit; HDL‐C, high‐density lipoprotein cholesterol; HGB, hemoglobin; LDL‐C, low density lipoproteincholesterol; LPa, lipoprotein a; MCH, mean corpuscular hemoglobin; MCHC, mean corpuscular hemoglobin contentration; MCV, mean corpuscular volume; NGAL, neutrophil gelatinase‐associated lipocalin; Phos, phosphorus; RBC, red blood cell; TC, total cholesterol; TG, Total triglyceride; TP, total protein; UA, uric acid .

### 3.2 Association of NGAL levels with clinical and biochemical parameters

We divided the CKD study population into five groups according to stages of eGFR as defined in Kidney Disease: Improving Global Outcomes for CKD.[26] The NGAL levels of each respective stage were: 157.6 ± 64.04 ng/mL, 228.3 ± 127.3 ng/mL, 346.9 ± 159.4 ng/mL, 552.9 ± 323.5 ng/mL, and 729.5 ± 282.7 ng/mL (*P* < 0.001; Table 1).

**Figure 1:**
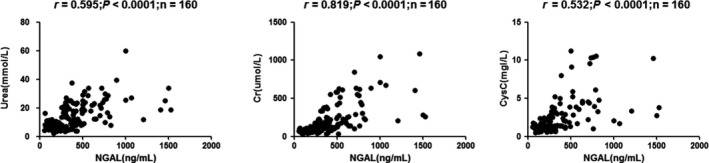
Correlation analyses among the tested parameters in chronic kidney disease (CKD) patients. Correlations were analyzed between (A) neutrophil gelatinase‐associated lipocalin (NGAL) and urea (*r* = 0.595, *P* < 0.0001), (B) NGAL and creatinine (Cr; *r* = 0.819, *P* < 0.0001), and (C) NGAL and cystatin C (CysC; *r* = 0.532, *P* < 0.0001).

**Figure 2:**
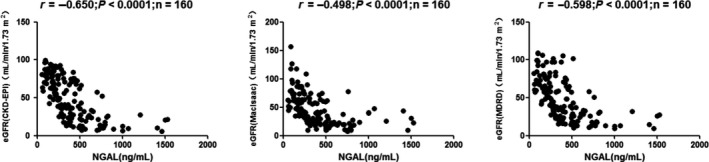
Correlation analyses among the tested parameters in chronic kidney disease (CKD) patients. Correlations were analyzed between (A) neutrophil gelatinase‐associated lipocalin (NGAL) and estimated glomerular filtration rate (eGFR; Chronic Kidney Disease Epidemiology Collaboration; *r* = −0.650, *P* < 0.0001), (B) NGAL and eGFR (MacIsaac; *r* = −0.498, *P* < 0.0001), and (C) NGAL and eGFR (modification of diet in renal disease [MDRD]; *r* = −0.598, *P* < 0.0001).

**Figure 3:**
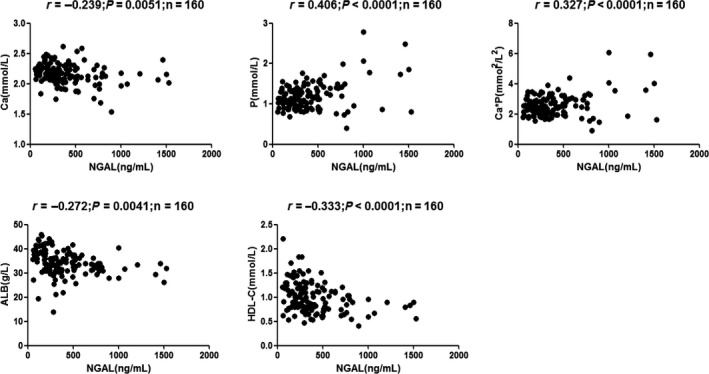
Correlation analyses among the tested parameters in chronic kidney disease (CKD) patients. Correlations were analyzed between (A) neutrophil gelatinase‐associated lipocalin (NGAL) and calcium (Ca) (*r* = −0.239, *P* = 0.0051), (B) NGAL and phosphorus (P) (*r* = 0.406, *P* < 0.0001), (C) NGAL and calcium*phosphorus (Ca*P) (*r* = 0.327, *P* < 0.0001), (D) NGAL and albumin (ALB; *r* = −0.272, *P* = 0.0041), and (E) NGAL and high‐density lipoprotein cholesterol (HDL‐C; *r* = −0.333, *P* < 0.0001).

**Figure 4:**
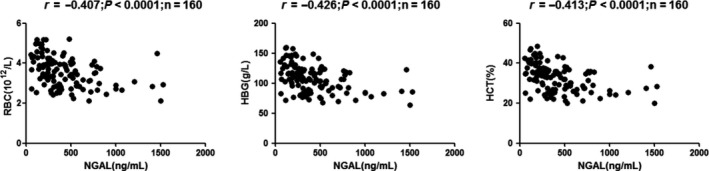
Correlation analyses among the tested parameters in chronic kidney disease (CKD) patients. Correlations were analyzed between (A) neutrophil gelatinase‐associated lipocalin (NGAL) and red blood count (RBC; *r* = −0.407, *P* < 0.0001), (B) NGAL and hemoglobin (HGB) (*r* = −0.426, *P* < 0.0001), and (C) NGAL and hematocrit (HCT) (*r* = −0.413, *P* < 0.0001)

In univariate analysis, sNGAL correlated positively with urea, CysC, and Cr, but inversely with eGFR: urea (*r* = 0.595, *P* < 0.0001), Cr (*r* = 0.819, *P* < 0.0001), and CysC (*r* = 0.532, *P* < 0.0001; Figure 1); eGFR (CKD‐EPI; *r* = −0.650, *P* < 0.0001), eGFR (MacIsaac; *r *= −0.498, *P* < 0.0001), and eGFR (modification of diet in renal disease [MDRD]; *r* = −0.598, *P* < 0.0001; Figure 2); Ca (*r* = −0.239, *P* = 0.0051), P (*r* = 0.406, *P* < 0.0001), Ca*P (*r* = 0.327, *P* < 0.0001), ALB (*r* = −0.272, *P* = 0.0041), and HDL‐C (*r* = −0.333, *P* < 0.0001; Figure 3); RBC (*r* = −0.407, *P* < 0.0001), HGB (*r* = −0.426, *P* < 0.0001), and HCT (*r* = −0.413, *P* < 0.0001; Figure 4).

On the contrary, no significant correlation was found between NGAL and other parameters, such as sex, uric acid (UA), total cholesterol (TC), total triglyceride (TG), or total protein (TP) (*P* > 0.05); however, the age group of CKD G1 showed statistical difference with the other groups and this may be because of renal aging among the normal elderly population.

### 3.3 NGAL in the Kidney Failure Risk Equation categories of CKD

**Figure 5:**
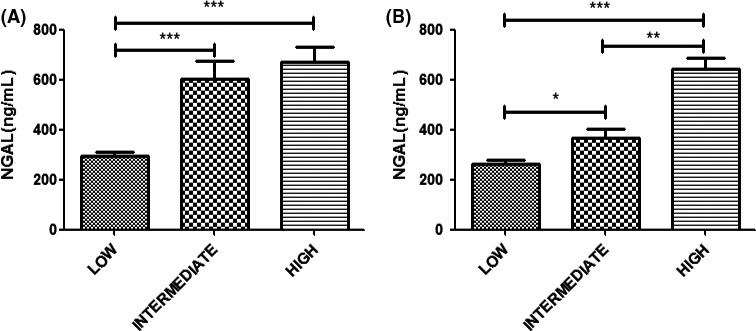
Levels of neutrophil gelatinase‐associated lipocalin (NGAL) at the (A) 2‐ and (B) 5‐year risk of end‐stage renal disease using Kidney Failure Risk Equations. *P < 0.05, **P < 0.001, ***P < 0.0001.

The Kidney Failure Risk Equation (KFRE), a tool for predicting the risk of ESRD, has also been recommended by the 2016 Clinical Practice Guideline on management of older patients with CKD.[6] The KFRE uses a simple online application (http://kidneyfailurerisk.com), which requires four variables: patient age, sex, GFR, and ALB:Cr ratio, to estimate a percentage probability of reaching end stage in 2 and 5 years. The levels of risk can be identified and grouped into low risk, intermediate risk, and high risk (Figure 5).[27]

The sNGAL levels at the 2‐year risk of ESRD showed a significant statistical difference between the low‐ and intermediate‐risk categories (*P* < 0.001) and between the low‐ and high‐risk categories (*P* < 0.001), but no statistical difference between the intermediate‐ and high‐risk categories (*P* > 0.05). The concentrations of NGAL in the three risk categories were 294.9 ± 185.9 μg/L (low‐risk), 600.7 ± 344.2 μg/L (intermediate‐risk), and 671.7 ± 304.9 μg/L (high‐risk), respectively. The NGAL levels at the 5‐year risk of ESRD showed a significant statistical difference among the three categories (*P* < 0.05). The concentrations of sNGAL in the three risk categories were 262.4 ± 154.5 μg/L (low‐risk), 368.5 ± 120.7 μg/L (intermediate‐risk), and 644.0 ± 321.5 μg/L (high‐risk), respectively (Figure 5).

### 3.4 Diagnostic performance

ROC analysis was used to evaluate the diagnostic performance of the four tested markers for CKD and demonstrated that the four markers showed a similar diagnostic meaning or AUC: 0.9870, 0.9939, 0.8651, and 0.9055 for NGAL, CysC, urea, and Cr, respectively (Table 2).

**Table 2:** Diagnostic performance of NGAL, cystatin C, urea and Cr to confirm the eGFR of total patients

Parameters	AUC	95% CI	*P*
NGAL	0.987	0.9768‐0.9971	<.0001
CysC	0.9939	0.9893‐0.9985	<.0001
Urea	0.8651	0.8263‐0.9039	<.0001
Cr	0.9055	0.8748‐0.9361	<.0001

Abbreviations: Cr, creatinine; CysC, cystatin C; NGAL, neutrophil gelatinase‐associated lipocalin.

### 3.5 Cutoff NGAL value in GFR categories of CKD

The cutoff value of urine NGAL was 235.7 mg/L for Stage 2, 249.45 mg/L for Stage 3, 305.8 mg/L for Stage 4, and 422.64 mg/L for Stage 5. The cutoff value of serum NGAL (sNGAL) was highly accurate for the AUC > 0.85 among the four stages (Table 3).

**Table 3:** Cutoff values of the NGAL in the eGFR categories of CKD2‐5

Parameter	Stages	AUC	95% CI	Sensitivity (%)	Specificity (%)	Youden index	Cutoff value (μg/L)
NGAL	2	0.857	0.780‐0.934	0.725	0.9	0.625	235.7
	3	0.869	0.812‐0.925	0.897	0.726	0.623	249.45
	4	0.892	0.845‐0.939	0.898	0.69	0.602	305.8
	5	0.897	0.848‐0.948	0.923	0.752	0.675	422.64

Abbreviation: NGAL, neutrophil gelatinase‐associated lipocalin.

## 4 DISCUSSION

The main findings from the present study clearly indicate that NGAL represents a novel risk marker of kidney function decline in patients aged >50 years and >65 years with advanced CKD. Additionally, NGAL may be a predictive factor of risk stratification for reaching ESRD.

Aging increases the intrinsic renal susceptibility to injury with less recovery and regeneration ability. Microscopic changes accompanying renal aging include kidney weight, size reduction, and cortical atrophy.[28] Functional changes are manifested by GFR, effective renal plasma flow decline, and renal vascular resistance rising. Tubular cell apoptosis increased more significantly in aged rats after renal ischemia reperfusion.[29]

Acute kidney injury and CKD increase risk of ESRD among the elderly and the incidence increases even more after age 50 years. (Data from Chinese Research Data Services, 2015). Diabetes mellitus and hypertension are common diseases in the elderly and are proven to be two of the most important risk factors for kidney injury.[30] Renal aging plays as a crucial role in acute kidney injury and CKD, which is proven to be an interconnected syndrome.[31] Naudé et al found that plasma NGAL concentrations were significantly associated with age.[32]

NGAL, a ubiquitous 25‐kDa lipocalin iron‐carrying protein,[33]is originally isolated from neutrophils.[34] Researchers then found that it is also expressed in tissues, such as the kidney, liver, epithelial cells,[35, 36] and vascular cells in atherosclerotic plaques.[37] It came to the attention of clinical scientists because it was found to be one of the earliest, most robustly induced genes and proteins in the tubular epithelium of the distal nephron and released from tubular epithelial cells following tissue damage, such as ischemic renal injury.[38] Further studies found the dimeric molecular form from urine is originated from kidney tubular epithelial cells, and the monomeric form is produced by neutrophils.[39] This difference has the potential to improve the specificity of NGAL as a renal biomarker.

The NGAL showed good correlation with eGFR, CysC, Cr, and urea. Median concentrations of the four markers—NGAL, CysC, Cr, and Urea—progressively increased across stages of CKD as defined by the revised CKD guidelines. The four markers demonstrated similar diagnostic performance and accuracy for identifying renal function; especially, NGAL showed better AUCs than Cr and urea.

In this study, there was a correlation between NGAL and the respective clinical and biochemical parameters: RBC, HGB, HCT, Ca, P, Ca*P, ALB, and HDL‐C reflect NGAL taking part in a special underlying pathophysiology in CKD.

Our study confirmed the correlation between NGAL and anemia, with similar results to those of Jolanta’s study in the elderly.[40] Anemia is a common symptom of CKD. Furthermore, several systemic diseases, such as chronic inflammation and cancer, are associated with the presence of secondary anemia. Significant high‐circulating NGAL levels are another common feature of the above diseases. This may suggest a further, important exploration of the relation between anemia and NGAL. Many experiments have demonstrated that NGAL takes part in the physiology and pathophysiology of red blood cells. A recent study found that in vitro culture NGAL induces apoptosis and inhibits differentiation of erythroid progenitor cells, confirming NGAL as having a key role in anemia.[41] Another study considered that NGAL may be an attractive therapeutic target for anemia under certain pathological conditions.[42]

We found respective correlations between NGAL and Ca, P, Ca*P, ALB, and HDL‐C, which showed that NGAL may participate in the calcium and phosphorus metabolic disorders and malnutrition of CKD. Older patients often suffer from malnutrition and hypoalbuminemia. Patients with CKD are at a substantial risk for malnutrition, characterized by protein energy wasting and micronutrient deficiency, so older patients with CKD are more susceptible to suffering from malnutrition.

Inflammation and reactive oxygen species play important roles in the malnutrition of CKD.[43, 44, 45, 46] In addition to being a marker for the progression of CKD,[47, 48, 49]NGAL was initially found to be released by circulating neutrophils and was recently supported as an inflammatory marker.[50]A recent study found that serum NGAL levels correlate with the ALB detection index. Further experiments suggested that hypoalbuminemia may result from the presence of some undetected oxidized/modified ALB molecules, involved in a vicious cycle of systemic inflammation, reactive oxygen species, and neutrophil activation.[51]These provide a possible mechanism for NGAL participation in hypoalbuminemia.

Levels of 2‐ and 5‐year risk of ESRD can be identified and grouped into three categories. The data showed a significant difference between the three categories of 5‐year risk with NGAL. Though no significantly statistical difference was observed between the intermediate‐ and high‐risk category of 2 years, the concentration of NGAL showed obvious difference between the low‐ and the intermediate‐/high‐risk categories. The study indicated that NGAL could be used to predict the end stage of CKD risk levels. There has been evidence suggesting that NGAL may even be involved as a mediator of CKD progression; animal experiments in the same study showed that NGAL knockout mouse markedly reduced renal lesions seen in CKD progression.[52]

Our study has several limitations. First, it was an observation study, and as such, no mechanism experiments were involved. Second, serum NGAL was measured only at baseline. Follow‐up studies should be performed at every stage for further risk assessment on the end‐stage and dialysis in this population. Third, the number of patients was relatively low, and although we performed additional statistical procedures, this fact could have influenced the final results. Another limitation could be the fact that we did not assess urinary NGAL in our cohort, as it was recently established not to improve risk prediction of progressive CKD in several studies.[53, 54, 55, 56, 57]

## 5 CONCLUSION

To the best of our knowledge, this is the first study to evaluate the prognostic value of serum NGAL in older patients with CKD. We show that NGAL levels are independently associated with eGFR and renal‐disease‐related clinical parameters, predicting renal function decline. Further in‐depth examinations should be undertaken to verify whether these findings can be confirmed in a larger older population and to determine whether therapeutic measures targeting NGAL balance would be helpful in delaying the progression of CKD and clinical complications, such as anemia and hypoalbuminemia. Recently, a study has established a pivotal role for NGAL in regulating the progression of CKD and cyst by mediating the mitogenic effect of EGFR, consistent with its role in cell proliferation in cystogenesis.[52] More studies are needed to establish the mechanism of NGAL in the aging kidney.

## CONFLICTS OF INTEREST

Nothing to disclose.
